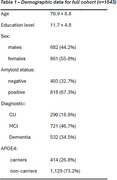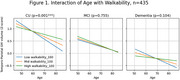# Associations of neighborhood walkability in Barcelona with cognitive function and gray matter volume in cognitively unimpaired older adults and patients with mild cognitive impairment and dementia

**DOI:** 10.1002/alz70860_104295

**Published:** 2025-12-23

**Authors:** Carmen M Colceriu, Alex López, Pablo Aguilar, Eleni Palpatzis, Judith Garcia‐Aymerich, Mark Nieuwenhuijsen, Adrià Tort‐Merino, María Franquesa‐Mullerat, Sara E Zsadanyi, Antonia Valentín, Sami Petricola, Juan Fortea, Alberto Lleo, Neus Falgàs Martínez, Raquel Sánchez‐Valle, Alexandre Bejanin, Eider M Arenaza‐Urquijo

**Affiliations:** ^1^ Global Health Institute Barcelona (ISGlobal), Barcelona, Spain; ^2^ University of Pompeu Fabra (UPF), Barcelona, Spain; ^3^ Sant Pau Memory Unit, Department of Neurology, Hospital de la Santa Creu i Sant Pau, Institut d'Investigació Biomèdica Sant Pau (IIB SANT PAU), Facultad de Medicina ‐ Universitat Autònoma de Barcelona, Barcelona, Spain; ^4^ Alzheimer's Disease and Other Cognitive Disorders Unit, Neurology Department, Hospital Clinic, Barcelona, Spain; ^5^ Centro de Investigación Biomédica en Red de Fragilidad y Envejecimiento Saludable (CIBERFES), Madrid, Spain

## Abstract

**Background:**

Physical activity has been associated with preserved executive functions and brain health in aging. A potential element promoting physical activity is neighborhood walkability – a measurement of how friendly an area is to walk. We evaluated the associations of walkability with executive functions and gray matter volumes and its interaction with age in cognitively unimpaired (CU) older adults and patients with mild cognitive impairment (MCI) and dementia.

**Method:**

We included 1543 participants from UBRAIN study recruited in Clinic and Sant Pau Hospitals (Barcelona, Spain) ‐18.8% CU, 46.7% MCI, and 34.5% dementia (55% Alzheimer's Disease, 20.5% Frontotemporal dementia, 14.5% Lewy body, other 10%, Table 1). Executive functions (Trail Making Test and WAIS Digits forward and backward) and MRI‐based gray matter (GM) volumes in temporo‐parietal regions including medial temporal structures, were considered as outcomes. The walkability index included 8 indices (eg. street connectivity, slopes) assessed within a 100m radius around participant residences. We ran linear regression models, both in the full sample and in CU, and in patients with MCI and dementia separately, adjusted by sex, age and education and evaluated the interaction of walkability with age.

**Result:**

In the full sample, there was a main effect of walkability on WAIS Digits forward scores (β=3.774, *p* <0.001) such that living in more walkable areas was associated with better performance, but no main effect on GM volume or interactions with age was observed. When stratified by the diagnostic group, the main effect was observed only in CU (β=5.429, *p* = 0.01) and patients with dementia (β=5.448, *p* = 0.025), but not in MCI participants (*p* = 0.345). In CU, higher walkability counteracted the effect of age on temporo‐parietal GM volumes (β=2.84, *p* = 0.001, Figure 1).

**Conclusion:**

Living in more walkable areas was associated with better attention in both CU and patients with dementia and attenuated the effect of age on brain structure in CU. Walkability may be a protective factor in aging and dementia. Further research is needed to determine whether this effect operates through increased physical activity.